# Low cytomolecular diversification in the genus *Stylosanthes* Sw. (Papilionoideae, Leguminosae)

**DOI:** 10.1590/1678-4685-GMB-2018-0250

**Published:** 2020-03-06

**Authors:** Ana Luiza Franco, Amanda Figueredo, Lívia de Moraes Pereira, Saulo Marçal de Sousa, Gustavo Souza, Marcelo Ayres Carvalho, Marcelo F. Simon, Lyderson Facio Viccini

**Affiliations:** 1 Universidade Federal de Juiz de Fora, Departamento de Biologia, Laboratório de Genética, Juiz de Fora, MG, Brazil.; 2 Universidade Federal de Pernambuco, Departamento de Botânica, Laboratório de Citogenética e Evolução Vegetal, CCB, Recife, PE, Brazil.; 3 Empresa Brasileira de Pesquisa Agropecuária, Embrapa Cerrados, Brasília, DF, Brazil.; 4 Empresa Brasileira de Pesquisa Agropecuária, Embrapa Recursos Genéticos e Biotecnologia, PqEB, Brasília, DF, Brazil.

**Keywords:** Arachis, cytogenetics, evolution, Leguminosae, Stylosanthes

## Abstract

*Stylosanthes* (Papilionoideae, Leguminosae) is a predominantly Neotropical genus with ~48 species that include worldwide important forage species. This study presents the chromosome number and morphology of eight species of the genus *Stylosanthes* (*S. acuminata, S. gracilis, S. grandifolia, S. guianensis, S. hippocampoides, S. pilosa, S. macrocephala*, and *S. ruellioides*). In addition, staining with CMA and DAPI, *in situ* hybridization with 5S and 35S rDNA probes, and estimation of DNA content were performed. The interpretation of *Stylosanthes* chromosome diversification was anchored by a comparison with the sister genus *Arachis* and a dated molecular phylogeny based on nuclear and plastid loci. *Stylosanthes* species showed 2*n* = 20, with low cytomolecular diversification regarding 5S rDNA, 35S rDNA, and genome size. *Arachis* has a more ancient diversification (~7 Mya in the Pliocene) than the relatively recent *Stylosanthes* (~2 Mya in the Pleistocene), and it seems more diverse than its sister lineage. Our data support the idea that the cytomolecular stability of *Stylosanthes* in relation to *Arachis* could be a result of its recent origin. The recent diversification of *Stylosanthes* could also be related to the low morphological differentiation among species, and to the recurrent formation of allopolyploid complexes.

## Introduction

Chromosomal evolution, including polyploidization (auto or allopolyploidy), chromosomal rearrangements, and heterochromatin polymorphisms are known to be important mechanisms promoting speciation in plants (Raskina *et al.*, 2008; Soltis and Soltis, 2009; Reis *et al.*, 2014; Pinto *et al.*, 2016). However, chromosome changes may be neutral, with no direct impact on the adaptability or reproductive viability of the individuals carrying the polymorphism (Levin, 2002), and will not necessarily result in speciation. For example, Pimentel *et al.* (2017) investigated patterns of diversification and chromosomal evolution in Pooideae (Poaceae) in the light of past environmental changes. In this group, the haploid basic chromosome number has remained stable, with no direct association of chromosome transitions with diversification shifts.

One aspect that would influence the accumulation of cytomolecular diversity in plant lineages over time is the age of the group (Levin, 2002; Soltis and Soltis, 2009; Susek *et al.*, 2016). Therefore, it would be expected that groups of recent diversification would have more stable and uniform karyotypes, while those with older divergences would have time to accumulate more cytomolecular differences, resulting in higher karyotypical diversity. However, it is not clear if the timing of diversification of a given lineage reflects variation at the cytogenetic level. For example, there are cases of recently evolved lineages with highly variable karyotypes (e.g., genus *Nothoscordum* [Amaryllidaceae] Souza *et al.*, 2012), and ancient groups showing high karyotype stability (e.g., subfamily Bombacoideae [Malvaceae] Costa *et al.*, 2017).

In recent years, different studies reported new insights on the phylogeny and karyotype evolution of legumes, making this group an interesting model to investigate diversification and karyotype evolution. The period between the origin and diversification of Leguminosae was estimated to be short, with the divergence of the main lineages around 60 million years ago (Mya) (Lavin *et al.*, 2005). Within the Leguminosae, subfamily Papilionoideae comprises one of the most diverse and ecologically successful plant radiations, presenting a diversification history stemming from the early Cenozoic (Lavin *et al.*, 2005; Cardoso *et al.*, 2012; LPWG - Legume Phylogeny Working Group, 2017). Wong *et al.* (2017) suggested that a single whole genome duplication (WGD) event near to the base of Fabales was associated with the onset of Leguminosae diversification and that subsequent chromosome number reductions contributed to the success and diversification of Papilionoideae.

Within the papilionoid Pterocarpus clade (Cardoso *et al.*, 2013), the sister genera *Arachis* and *Stylosanthes* represent a useful model for analyzing the evolution of karyotypic diversity in closely related lineages of similar size. The South American genus *Arachis* comprises approximately 82 species arranged in nine sections that were grouped according to the geographic distribution, morphology, and cross-compatibility (Krapovickas and Gregory 1994; Valls and Simpson, 2005), and includes widely cultivated crops (peanut) and forages. *Stylosanthes* is a predominantly neotropical genus with nearly 48 species with low inter and intraspecific morphological variation (Stace and Cameron, 1984; Costa, 2006), including important species cultivated as forage in Africa and Australia (Costa, 2006). Due to their adaptability to low fertility soils and nitrogen fixing capacity, some species of the genus are grown to recover degraded soils (Sheltom *et al.*, 2005; Starr *et al.*, 2013; Liu *et al.*, 2016).

The species of Arachis are mostly autogamous and have the basic chromosome number x = 10 (except x = 9 in A. decora Krapov., W.C.Greg. & Valls, A. porphyrocalyx Valls & C.E.Simpson, A. palustris Krapov., W.C.Greg. & Valls, and A. praecox Krapov., W.C.Greg. &Valls). There are diploid (2*n* = 2*x* = 20 or 2*n* = 2*x* = 18) and tetraploids species (2*n* = 4*x* = 40) with six different genomes (A, B, D, F, G, and K) (Fernández and Krapovickas, 1994; Peñaloza and Valls, 2005; Silvestri *et al.*, 2015; Ortiz *et al.*, 2017). DNA contents are known for 23 species of *Arachis*, with an average of 2C = 2.83 pg (http://data.kew.org/cvalues). On the other hand, *Stylosanthes* karyotype data are extremely scarce, with most data coming from chromosome counting, and few species with genome size and molecular cytogenetics were investigated (Vieira *et al.*, 1993; Chandra and Kaushal, 2009; Lira, 2015; Marques *et al.*, 2018). Most of the species of the genus are diploids (2*n* = 20) but few polyploid species (2*n* = 40, 60) were reported (Cameron, 1967; Karia, 2008). Their chromosomes are small and with similar morphology, making it difficult to recognize chromosome pairs and to interpret the karyotype evolution (Vieira *et al.*, 1993). A recent study based on cytogenetic and genomic data of the allopolyploidy *S. scabra* (2n = 40) revealed its origin from two diploid progenitors (Marques *et al.*, 2018).

Phylogenetic and cytogenetic analyses are useful to investigate the *time and mode of* genome evolution, as well as to examine the impact of chromosome changes in plant diversification (e.g., Escudero *et al.*, 2012). The combination of fluorescent *in situ* hybridization (FISH) and/or fluorochrome banding with phylogenetic comparative methods is a powerful tool for reconstructing detailed karyotype evolution (Guerra, 2012; Van-Lume *et al.*, 2017). These techniques are useful to display chromosome morphological features, heterochromatin distribution, and physical locations of repetitive DNA in plants. Such combination of methods is particularly interesting in revealing relationships among species and their genomic organizations, helping to understand the evolutionary history of plant groups (Reis *et al.*, 2014; Ortiz *et al.*, 2017; Samoluk *et al.*, 2017). Here, we analyzed chromosome numbers and morphology of eight species of *Stylosanthes*, including chromomycin A3 (CMA) and 4’-6-diamidino-2-phenylindole (DAPI) chromosome banding, FISH for 5S and 35S ribosomal DNA (rDNA), as well as genome size estimate by flow cytometry. These data were used to investigate the relationship between timing of diversification and karyotypic diversity, using for comparison the sister genus *Arachis*, which has been extensively characterized in terms of cytology. The interpretation of chromosome variation within genera was anchored by a dated molecular phylogeny based on nuclear and plastid sequences.

## Materials and Methods

### Plant material

The present study was based on material obtained from seeds of 12 accessions of eight *Stylosanthes* species [*S. acuminata*, *S. gracilis*, *S. grandifolia*, *S. guianensis*, *S. hippocampoides*, *S. macrocephala*, *S. pilosa*, and *S. ruellioides*]. Five accessions of *S. guianensis* (1480, 4171, 1463, LC2538, and cv. Mineirão) were also investigated (see Table S1).

For cytogenetic analysis, root tips obtained from seeds or seedlings were pretreated with 8-hydroxyquinoline (0.003 M) for 7 h at room temperature, fixed in ethanol:acetic acid (3:1; v/v) for 24 h at room temperature, and then stored at -20 °C. Fixed root tips were washed in distilled water and digested in a solution of 2% (w/v) cellulase /20% (v/v) pectinase (Onozuka) at 37 °C for 5 h. The slides were prepared according to Carvalho and Saraiva (1993, 1997).

### Chromosome banding

Chromosome banding was performed according to Schweizer (1976) with few modifications. Slides were stained with CMA (0.5 mg/mL) for 1h, dystamicyn (0.1 mg/mL) for 30 min and DAPI (2 μg/mL) for 30 min. The slides were mounted in glycerol:McIlvaine buffer pH 7.0 (1:1 v/v), and examined using an epifluorescence microscope (Olympus BX51).

### Fluorescent *in situ* hybridization (FISH)

Fluorescent *in situ* hybridization (FISH) was performed using a 18S (located in a repetitive unit known as 45S rDNA, Roa and Guerra, 2015; or 35S rDNA, Garcia *et al.*, 2017) spacer rDNA probes from *Triticum aestivum* L. (Gerlach and Bedbrook, 1979) and 5S rDNA probes from *Zea mays* L. (courtesy of Koo and J. Jiang). Each probe was labeled with digoxigenin by nick translation using DIG-Nick Translation Mix (Roche), and then hybridized according to Jiang *et al.* (1995) with some modifications. The hybridization mixture was denatured at 90 °C for 10 min and immediately transferred to an icebox. The slides were denatured at 85 °C for 1 min and treated with a series of alcohol washes (70% ice cold, 90% and 100% at room temperature for 5 min each). The hybridization mixture was then added to the slides and the chromosomes allowed to hybridize at 37 °C for 24 h. Post hybridization washes were carried out using 2 SSC buffer (0.3 mol/L sodium citrate, 0.03 mol/L sodium chloride, pH 7) and 1 PBS buffer (0.136 mol/L sodium chloride, 0.27 mol/L potassium chloride, 0.1 mol/L dibasic sodium phosphate, 0.2 mol/L monobasic potassium phosphate, pH 7.4). Probes were detected with anti-DIG conjugate with rhodamine (Sigma) and post detection washes were performed using 1 TNT buffer (0.1 mol/L Tris, 0.15 mol/L sodium chloride, 0.05% Tween-20) and 1X PBS at room temperature. Metaphases were counter-stained with 2 μg/mL of DAPI (Sigma). The slides were mounted in Vectashield (Vector, Burlingame, California, USA), and samples were rehybridized. Images with 5S (red) and 18S (green pseudocolor) signals were merged using Adobe Photoshop CS5.

### Flow cytometry

Nuclear DNA content estimation was performed according to Galbraith *et al.* (1983) using a FacsCanto II Flow Cytometer. Approximately 20-30 mg of young and fresh leaves and the same amount of tissue of standard references (*Pisum sativum* L. 9.09 pg) were chopped with 1 mL of WPB lysis buffer (Loureiro *et al.*, 2007). The histograms were analyzed with Flowing Software 2.5.

DNA nuclear content (pg) of each sample was estimated by the relative fluorescence intensity of the sample and the internal reference standard. Three samples of each species/accessions were measured according to Dolezel *et al.* (2003):

$$DNA_{content}=\frac{PIFI\;of\;sample\times DNA\;content\;of\;standard}{PIFI\;of\;standard}$$

were PIFI is the fluorescence intensity of propidium iodide in G1 cells. DNA content among *S. guianensis* accessions was analyzed by ANOVA using Genes computational software (Cruz, 2013).

### Phylogenetic sampling

The phylogenetic analysis of *Stylosanthes* and its sister genus *Arachis* was based on ITS nuclear region (ITS1-5.8S-ITS2) and *mat*K plastid gene. ITS and *mat*K sequences of 25 species of *Arachis* were retrieved from previous studies (see GenBank references in Table 1; Lavin *et al.*, 2001; Bechara *et al.*, 2010). However, only four *matK* sequences were available for *Arachis* species. New ITS and *matK* sequences for ten accessions of *Stylosanthes* were generated (Genbank accession numbers MH223599 - MH223618). The related species *Chapmannia floridana* Torr. and A. Gray (Cardoso *et al.*, 2013) was included as an outgroup (Genbank accession numbers KJ772648 and AF203562).

**Table 1:** Species analyzed with their respective chromosome numbers, DNA content (average and coefficient of variation), and GenBank accession numbers.

Genus / Species	2n	2C-value (pg)	CV (%)	nº of rDNA sites		GenBank No.		References	
				5S	35S	ITS	*mat*K	C-values	rDNA
***Arachis*** **L.**									
*A. batizocoi* Krapov. *&* W.C.Greg.	20	2.83	3.65	1	3	AY615256.1	-	Samoluk *et al.,* 2014	Robledo and Seijo 2010
*A. cardenasii* Krapov. *&* W.C.Greg.	20	3.01	4.15	1	4	AY615236.1	-	Samoluk *et al.,* 2014	Robledo *et al.*, 2009
*A. correntina* (Burkart) Krapov. *&* W.C.Greg.	20	2.85	3.55	1	2	AF203554.1	-	Samoluk *et al.,* 2014	Seijo *et al.,* 2004
*A. cruziana* Krapov., W.C. Greg. *&* C.E. Simpson	20	2.59	4.33	3	3	AY615259.1	-	Samoluk *et al.,* 2014	Robledo and Seijo, 2010
*A. duranensis* Krapov. *&* W.C.Greg.	20	2.55	3.76	1	2	AY615240.1	-	Samoluk *et al.,* 2014	Seijo *et al.,* 2004
*A. helodes* Krapov. *&* Rigoni	20	2.81	2.59	1	3	AY615241.1	-	Samoluk *et al.,* 2014	Robledo *et al.,* 2009
*A. hypogaea* L.	40	2.80	2.80	2	5	AF156675.2	KX257487.1	Samoluk *et al.,* 2014	Robledo and Seijo, 2010
*A. ipaensis* Krapov.	20	3.19	3.71	1	3	AY615257.1	-	Samoluk *et al.,* 2014	Robledo and Seijo, 2010
*A. kretschmeri* Krapov. *&* W.C.Greg.	20	-	-	1	1	AY615220.1	-	-	-
*A. kuhlmannii* Krapov. *&* W.C.Greg.	20	3.09	4.11	1	3	AY615219.1	-	Samoluk *et al.,* 2014	Robledo *et al.,* 2009
*A. magna* Krapov., W.C.Greg. *&* C.E.Simpson	20	3.22	3.70	1	4	AF203555.1	-	Samoluk *et al.,* 2014	Robledo and Seijo, 2010
*A. major* Krapov. *&* W.C.Greg.	20	-	-	-	-	AY615229.1	AF203597.1	-	-
*A. monticola* Krapov. *&* Rigoni	40	2.85	2.76	2	5	AY615239.1	-	Samoluk *et al.,* 2014	Robledo and Seijo, 2010
*A. pintoi* Krapov. *&* W.C.Greg.	20	5.95	-	1	2	AJ320397.1	AF203596.1	Singh *et al.,* 1996	Lavia *et al.,* 2011
*A. schininii* Krapov. Valls *&* C.E.Simpson	20	3.18	4.11	1	2	AY615248.1	-	Samoluk *et al.,* 2014	Robledo *et al.,* 2009
*A. simpsonii* Krapov. *&* W.C.Greg.	20	3.08	3.94	1	3	AY615247.1	-	Samoluk *et al.,* 2014	Robledo *et al.,* 2009
*A. stenosperma* Krapov.	20	2.96	3.36	1	3	AY615252.1	-	Samoluk *et al.,* 2014	Robledo *et al.,* 2009
*A. triseminata* Krapov. *&* W.C.Greg.	20	3.05	-	1	2	AF204233.1	AF203599.1	Singh *et al.,* 1996	Raina *et al.,* 1999
*A. valida* Krapov. *&* W.C.Greg.	20	3.16	4.12	1	4	AY615244.1	-	Samoluk *et al.,* 2014	Robledo and Seijo, 2010
*A. villosa* Benth.	20	3.04	2.94	1	2	AF203558.1	-	Samoluk *et al.,* 2014	Robledo *et al.,* 2009
*A. williamsii* Krapov. *&* W.C.Greg.	20	3.2	3.68	1	1	AY615255.1	-	Samoluk *et al.,* 2014	Seijo *et al.,* 2004
***Stylosanthes*** **Sw.**									
*S. acuminata* M.B.Ferreira *&* Sousa Costa	20	2.2	4.19	1	2	MH223618	MH223603	This study	This study
*S. gracilis* Kunth	20	2.5	2.5	1	1	MH223617	MH223604	his study	This study
*S. grandifolia* M.B.Ferreira *&* Sousa Costa	20	2.5	4.21	1	1	MH223609	MH223599	This study	This study
*S. guianensis* (Aubl.) Sw. 1463	20	2.81	4.66	1	1	MH223611	MH223606	This study	This study
*S. guianensis* (Aubl.) Sw. 1480	20	3.02	3.48	1	1	MH223613	MH223602	This study	This study
*S. guianensis* (Aubl.) Sw. 4171	20	3.07	4.11	1	1	MH223612	MH223607	This study	This study
*S. guianensis* (Aubl.) Sw. LC2538	20	-	-	1	1	-	-	-	-
*S. guianensis* (Aubl.) Sw. Mineirão	20	2.80	3.06	1	1	MH223610	MH223605	This study	This study
*S. hamata* (L.) Taub.	20	1.80	-	1	1	-	-	Marques *et al.,* 2018	Marques *et al.,* 2018
*S. hippocampoides* Mohlenbr	20	2.53	3.85	1	1	MH223614	MH223608	This study	This study
*S. macrocephala* M.B.Ferreira *&* Sousa Costa	20	2.05	3.16	1	1	MH223616	MH223601	This study	This study
*S. ruellioides* Mart. ex Benth.	20	-	-	1	1	MH223615	MH223600	-	This study
*S. scabra* Vogel	40	2.86	-	2	1	-	-	Marques *et al.,* 2018	Marques *et al.,* 2018
	20	-	-	-	-	-	-	-	-
*S. pilosa* M.B.Ferreira *&* Sousa Costa									
*S. viscosa* (L.) Sw.	20	1.36	-	1	1	-	-	Marques *et al.,* 2018	Marques *et al.,* 2018

### DNA extraction, polymerase chain reaction (PCR), and sequencing

DNA extraction was performed according to Doyle and Doyle (1987) using seed cotyledon/embryo tissue (30 mg per species). DNA quantification was done in a NanoDrop 2000c spectrophotometer (Thermo Scientific). The complete ITS region (ITS1-5.8S-ITS2), was amplified with universal primers ITS 4 and ITS 5 (White *et al.*, 1990). The maturase K (*mat*K) gene was also amplified using universal primers 1RKIM and 3FKIM (Bremer *et al.*, 2002). Amplifications were performed in 50 μL of reaction volume containing 200 ng of genomic DNA and final concentrations of 1 buffer, 0.3 mM MgCl, 0.2 mM DNTP, 0.1 μM of each specific primers, 1.25 U *Taq* DNA polymerase, 1 TBT and MiliQ water to complete the volume. ITS PCRs were incubated at 95 ºC for 5 minutes, followed by 35 cycles of 1 min at 95 ºC, 1 min at 55 ºC, 1 min at 72 ºC, and finally, 1 min at 72 ºC in a thermocycler (Applied Biosystems). *mat*K reactions were run using the same program with few adjustments: annealing temperature (56 ºC) and extension time (10 min. After visualizing the PCR products in agarose 1% gel, the products were purified by precipitation. Purified PCR products were sequenced in a 3500 Genetic Analyzer (Applied Biosystems).

### Phylogenetic analysis and character reconstruction

DNA sequences were analyzed, edited, and aligned using Geneious software (version 7.1.9). An incongruence length difference test (ILD) was made in PAUP* (40.b10) (Swofford, 2002) to determine the statistical significance of incongruence between the data partitions. Bayesian inference search was performed with the ITS1-5.8S-ITS2 + *mat*K concatenated alignment. Since *matK* sequences were missing for most *Arachis* species (see Table 1) we also performed a phylogenetic analysis with ITS only to compare with the topology of the concatenated alignment (Figure S1). The Bayesian inference search was performed using CIPRES Science Gateway (Miller *et al.*, 2010) using MrBayes 3.2.1 on XSEDE (Ronquist and Huelsenbeck, 2003) plugin, with 10,000,000 generations.

To interpret the evolution of cytomolecular diversification of the genus *Stylosanthes*, a molecular clock analysis was performed using BEAST v.1.8.0 (Drummond *et al.*, 2012a). We used the Aikake Criterion in Jmodeltest2 (Darriba *et al.*, 2012) to select the best evolutionary model, which identified the GTR+I+G model for both partitions. Analyses were run using an uncorrelated log normal relaxed clock and a Yule Process speciation model. Two independent runs of 20,000,000 generations each were performed, sampling every 1,000 generations. In order to verify the effective sampling of all parameters and assess convergence of independent chains, we examined their posterior distributions in Tracer v.1.6. (Rambaut *et al.*, 2014) and the MCMC sampling was considered sufficient at effective sampling sizes (ESS) higher than 200. After removing 25% of samples as burn-in, the independent runs were combined and a maximum clade credibility (MCC) tree was constructed using TreeAnnotator v.1.8.2. (Drummond *et al.*, 2012a). Divergence time between *Arachis* and *Stylosanthes* estimated by a previous study (13.8 ± 1.7 Mya; Lavin *et al.*, 2004) was used as a secondary calibration point using a normal prior.

The interpretation of karyotype data evolution of *Stylosanthes* and *Arachis* was done by plotting 5S rDNA, 35S rDNA, and genome size data on the dated phylogeny obtained. For this, the ancestral state reconstruction of cytomolecular characters (number of 5S and 35S rDNA) were performed in Mesquite v. 3.51 (Maddison and Maddison, 2018). The trace character history function was used with the 50% majority-rule consensus tree from the Bayesian inference analyses. The ancestral state was inferred using maximum parsimony, in which all changes are equally probable. The number of 5S and 35S rDNA sites was assumed as continuous data. Chromosome data of species were taken from the literature (Marques *et al.*, 2018) or from this work, and they were treated as an unordered, multistate character.

## Results

**Figure 1:**
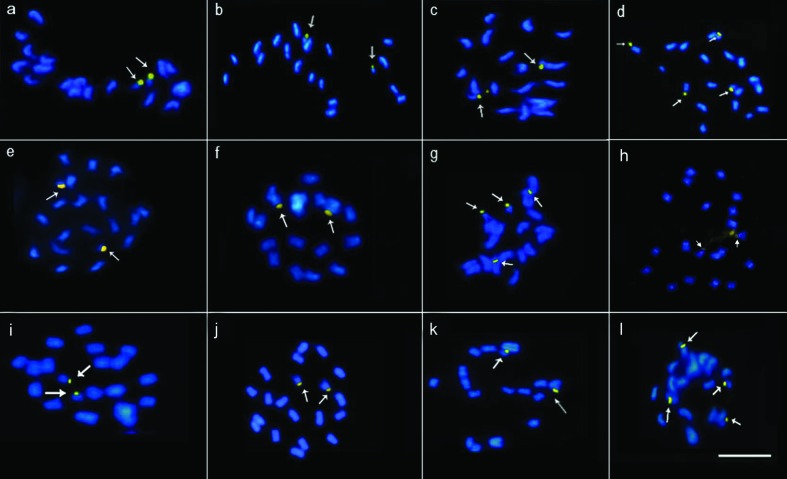
DAPI/CMA banding profile in *Stylosanthes* species. (a) *S. guianensis* 1480, (b) *S. guianensis* 4171, (c) *S. guianensis* 1463, (d) *S. acuminata,* (e) *S. guianensis* LC2538, (f) *S. pilosa*, (g) *S.guianensis* cv. Mineirão, (h) *S. ruellioides*, (i) *S. gracilis*, (j) *S. macrocephala*, (k) *S. hippocampoides*, (l) *S. grandifolia.* Bar = 10μm.

All species of *Stylosanthes* here investigated were diploids (2*n* = 20) with predominantly metacentric chromosomes, measuring on average 2.7 μm (Figures 1 [Fig f2] [Fig f3]-4). Most of the species (*S. hippocampoides*, *S. gracilis, S. macrocephala, S. pilosa, S. ruellioides*, and *S. guianensis* accessions 1480, 1463, 4171, and LC2538)*,* showed two CMA^+^/DAPI^-^ signals in the short arms of the smaller chromosomal pair. In addition to those bands, in *S. ruellioides* CMA^-^/DAPI^+^ proximal bands were also observed in all chromosomes (Figure 1). On the other hand, *S. acuminata*, *S. grandifolia*, and *S. guianensis* cv. Mineirão showed four CMA^+^ bands, two of them in the short arms of the smaller chromosome pair and the other two in the proximal region of a large chromosome pair (Figure 1d, g, l).

**Figure 2 f2:**
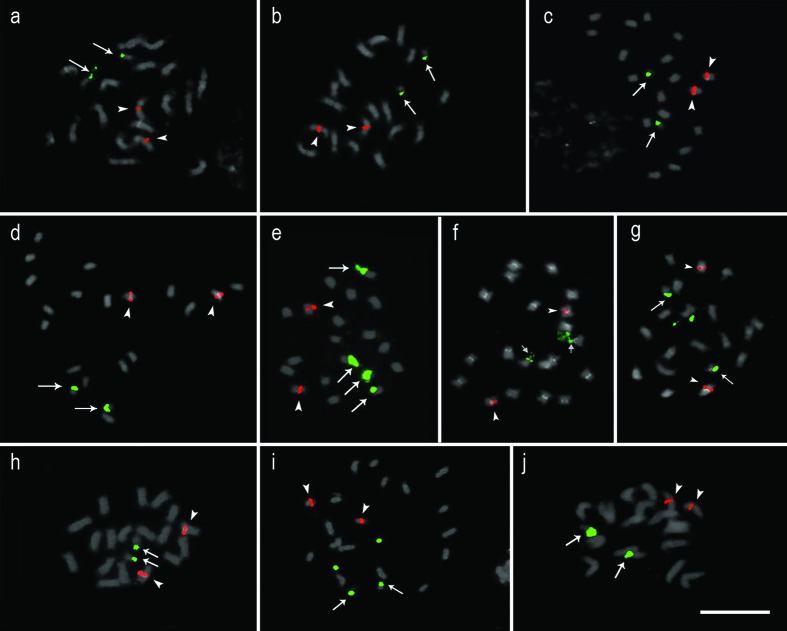
5S (red) and 35S (green) probes mapped in *Stylosanthes* species by fluorescent *in situ* hybridization. (a) *S. hippocampoides*, (b) *S. gracilis*, (c) *S. grandifolia*, (d) *S. macrocephala*, (e) *S. acuminata*, (f) *S. ruellioides*, (g) *S. guianensis* LC2538, two extra points are distended satellites, (h) *S. guianensis* cv. Mineirão, (i) *S. guianensis* 4171, two extra points are distended satellites, (j) *S. guianensis* 1480 Bar = 10μm.

**Figure 3 f3:**
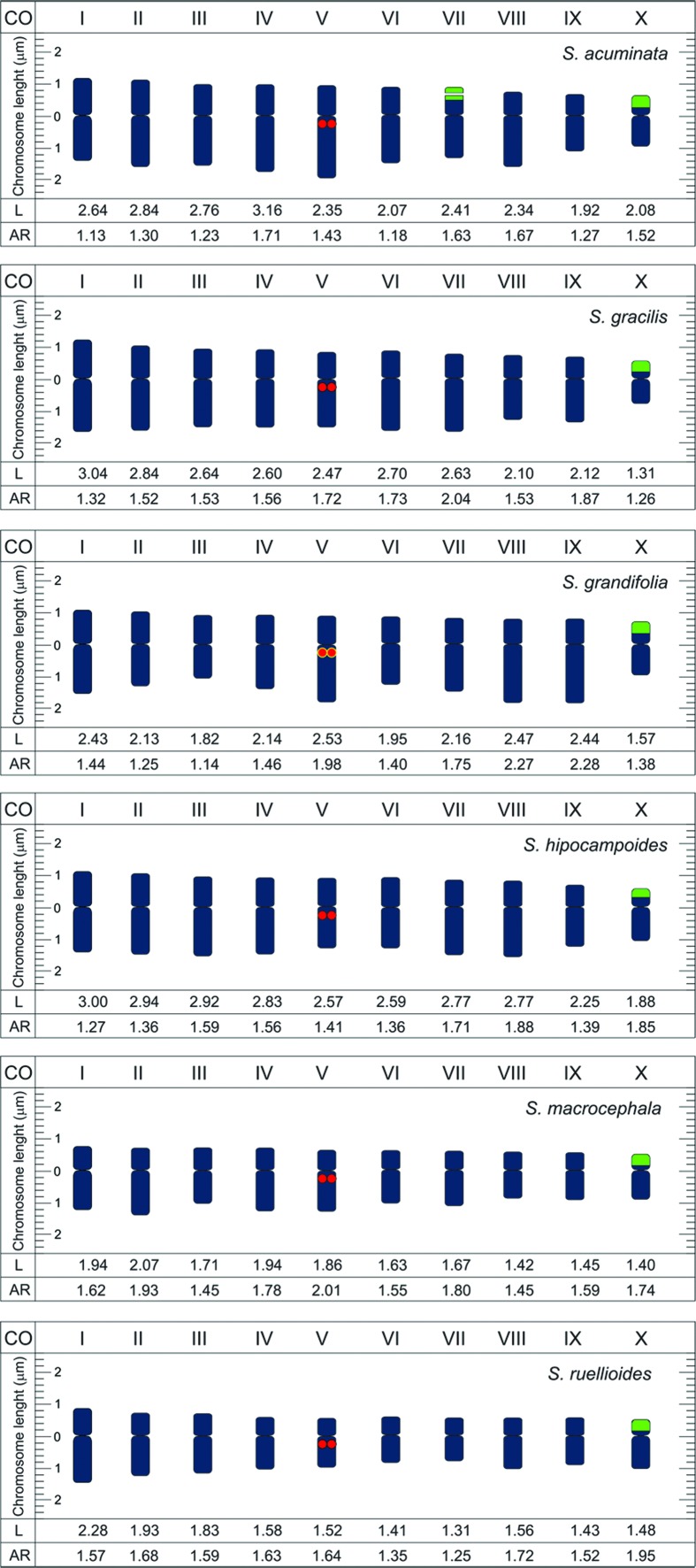
Idiograms of *Stylosanthes* species showing chromosome length (L), arm ratio (AR), 5S (red), CMA bands colocalized with 35S r DNA sites (green), and CMA bands colocalized with 5S (yellow).

**Figure 4:**
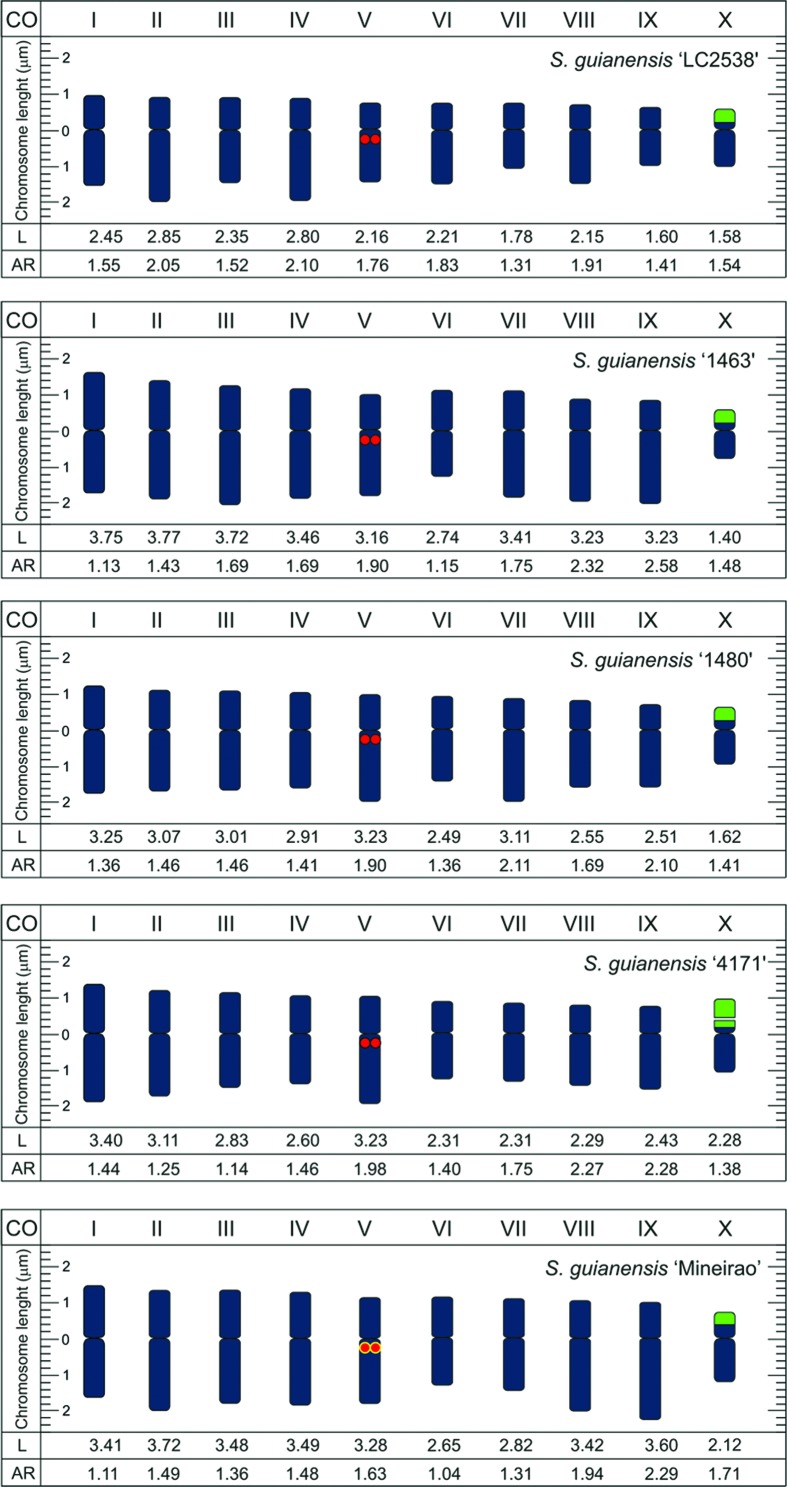
Idiograms of *S. guianensis* accessions showing chromosome length (L), arm ratio (AR), 5S (red), CMA bands co-localized with 35S r DNA sites (green), and CMA bands co-localized with 5S (yellow).

FISH analyses revealed 35S rDNA sites co-localized with the CMA^+^ bands in the short arms of the smaller acrocentric pair, as it was observed in *S. gracilis, S. grandifolia*, *S. hippocampoides*, *S. macrocephala*, and *S. ruellioides* (Figure 2a-d, f and Figure 3). The 5S rDNA sites were localized in the proximal region of one chromosome pair (Figure 2a-j and Figure 3). In *S. acuminata* we observed four 35S rDNA sites (Figure 2e). No variation in the number of rDNA sites was observed among *S. guianensis* accessions, being possible to map one pair of 5S rDNA and one pair of 35S rDNA (Figure 2g-j and Figure 4).

The DNA content among *Stylosanthes* species ranged from 2.05 to 3.07 pg (Table 1), with *S. macrocephala* showing the lowest value (2.05 pg) and *S. guianensis* accessions the highest (2.8 to 3.07). These values were analyzed by ANOVA and there were no significant differences between accessions (p=10.6194).

**Figure 5:**
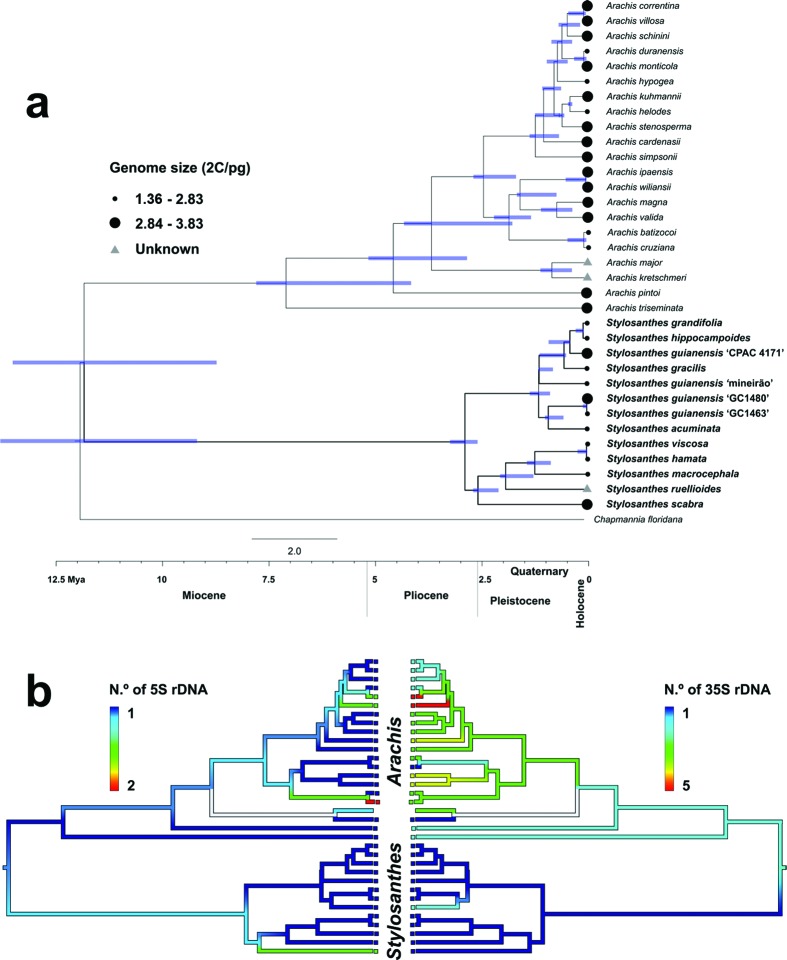
Chronogram of *Arachis* and *Stylosanthes* species based on BEAST analysis using the plastid *mat*K and nuclear ITS combined datasets. Blue bars indicate 95% highest posterior density intervals. **a**, comparative DNA content evolution, with symbols next to accessions proportional to genome size. **b**, ancestral character estimation of number of rDNA sites along the branches and nodes of the phylogeny. The color of edges in the tree represents observed and reconstructed values for chromosome number on the tree. Red colors correspond to relatively high numbers of rDNA sites; whereas dark blue colors represent low number of observed and reconstructed rDNA sites.

Molecular phylogenetic analysis including *Stylosanthes* (eight spp.) and *Arachis* (21 spp.) were performed. ILD test revealed no significant incongruence (p < 0.05) between *matK* and ITS datasets. Thus, Bayesian inference was conducted with the concatenated alignment *matK* + ITS since this alignment produced better resolved relationships. The concatenated alignment contains 1,180 base pairs, where the *mat*K region seems to be more conserved (identity = 98.3%) in both groups when compared to the ITS region (identity = 91.5%). The genera *Arachis* and *Stylosanthes* were recovered as monophyletic (Figure 5), with the diversification of *Arachis* starting on the upper Miocene (7.10 Mya; credibility interval 7.80–4.16 Mya), followed by a more recent origin of *Stylosanthes* at (2.90 Mya; CI 3.25–2.60 Mya). The clade represented by *S. acuminata*, *S. gracilis*, *S.grandifolia*, *S. hippocampoides*, and the four accessions of *S. guianensis* showed more recent diversification in the Pleistocene (~3 Mya).

Available cytogenetic data and genome size were also considered to interpret the molecular phylogenetic of the genera *Stylosanthes* and *Arachis* (Table 1, Figure 5). We categorized DNA contents into smaller (2C = 1.36 to 2.83 pg) and larger (2C = 2.84 to 3.28 pg). Most *Stylosanthes* fell into the 2C = 1.36–2.83 pg range size, except for two *S. guianensis* accessions (2C = 3.02 and 2C =3.07 pg). On the other hand, most *Arachis* species presented 2C values from 2C =2.87 to 3.28 pg, except for *A. duranensis*, *A. batizocoi*, *A. cruziana*, and *A. helodes* (2C = 2.55, 2.83, 2.59 and 2.81 pg, respectively) (Figure 5a). The number of 5S rDNA sites ranged from one to three in *Arachis*. All *Stylosanthes* species showed one pair of the rDNA 5S site. Regarding the 35S rDNA sites, it was possible to identify higher variation in *Arachis* (one to five pairs). For *Stylosanthes*, every species presented only one pair of the same site (except *S. acuminata*) (Figure 5b).

## Discussion

### Karyotype stability in *Stylosanthes*

Although most of the species of the genus *Stylosanthes* are diploids, few polyploid species, such as *S. hamata* and *S. scabra* with 2n= 40, and *S. erecta* with 2n=60 were reported (Cameron, 1967; Karia, 2008; Polido *et al.*, 2015). The diploid chromosome number (2*n* = 2*x* = 20) here observed is in agreement with previous report for the genus *Stylosanthes* (Vieira *et al.*, 1993; Lira, 2015; Marques *et al.*, 2018). Morphometric analysis of the *Stylosanthes* karyotypes revealed that the chromosomal profile was similar for all diploid species previously investigated (Vieira *et al.*, 1993), corroborating our findings. The predominance of metacentric chromosomes suggests a symmetrical karyotype, which is generally observed in other members of Leguminosae (Bandel, 1974; Kumari and Bir, 1989; Vieira *et al.*, 1993; Pinto *et al.*, 2016; Van-Lume *et al.*, 2017).

The stability or variability in legume karyotypes can be associated with the timing of diversification, reproduction strategies, and other factors related to the evolutionary history of each group (Soltis and Soltis, 2009; Gu *et al.*, 2016). Genome comparisons have showed conserved syntenic blocks between papilionoid genomes, especially among phylogenetically closely related species (Young and Bharti, 2012). In the genus *Phaseolus* (Papilionoideae), for example, a karyotypic stability of chromosomal numbers (except for dysploidy in the clade *Leptostachyus*) revealed by C-banding and fluorochrome staining (Zheng *et al.*, 1993; Almeida and Pedrosa-Harand, 2013) was observed and associated with a recent diversification over the last 5 Mya (Delgado-Salinas *et al.*, 2006). In this genus, the use of BAC-FISH mapping indicated a high level of macro-collinearity among homologous chromosomes (Almeida and Pedrosa-Harand, 2013; Fonsêca and Pedrosa-Harand, 2017). However, other legumes with recent diversification such as *Vigna* may present polymorphic karyotypes (Delgado-Salinas *et al.*, 2011; She *et al.*, 2014), indicating that time alone would not be the only factor responsible for the accumulation of karyotypic variability.[Bibr B113]

### Genome size in *Stylosanthes*

The small variation of DNA content (1.4 fold) in the *Stylosanthes* species analyzed here corroborates a scenario of karyotypic stability. Although the average of DNA estimation is in agreement with other estimations for the genus, we observed different C-values for two diploid species (*S. seabrana* B.L.Maass & ‘t Mannetje and *S. viscosa*) for which 5.45 pg were reported in the literature (Chandra and Kaushal, 2009). Variation of DNA content within a genus may be due to different factors such as recombination, deletion and retrotransposition (Piegu *et al.*, 2006; Baziz *et al.*, 2014). Among closely related species, other mechanisms appear to have some impact on genome size variations, such as expansion of tandem repeated DNA sequences, variation in intron size, and transfer of organellar DNA to the nucleus (Deutsch and Long, 1999; Morgante *et al.*, 2002; Adams and Palmer, 2003; Baziz *et al.*, 2014). Compared to other Leguminosae, the amount of DNA in *Stylosanthes* seems to be relatively high. *Leucaena macrophylla* Benth. has 2C value = 0.62 pg, *Trifolium arvense* L. was described with 2C = 0.78 pg and *Lotus coimbrensis* Willd. with 2C = 0.90 pg, although, *Lathyrus latifolius* L. (2C = 21.76 pg) and *Vicia faba* L. (2C = 54.8 pg) have the highest DNA content within the family (Cheng and Grant, 1973; Bennett *et al.*, 1982; Hartman *et al.*, 2000; Vizintin *et al.*, 2006). The reason why the DNA contents of eukaryotic genomes vary independently remains a matter of speculation. The same is true for the questions of whether there is a general tendency for increase or decrease of genome size and whether genome size and/or chromosome number have an adaptive value. Some authors hypothesized that three processes of genome evolution (shrinkage, expansion, and equilibrium) might be involved in achieving the optimal balance between genomic stability and plasticity (Gregory, 2001; Schubert and Giang, 2016).

### Heterochromatin pattern in *Stylosanthes*

The co-localization of all 35S rDNA sites with CMA^+^ bands as found in *Stylosanthes* species is commonly reported in plants (Siljak-Yakolav *et al.*, 2003). Interestingly, the co-localization of 5S rDNA with heterochromatic bands, as observed in *S. guianensis* cv. Mineirão and *S. grandifolia*, is a rare condition in other higher plants (Cabral *et al.*, 2006; Vasconcelos *et al.*, 2010; Bernardes *et al.*, 2013). Karyotype variability in Papilionoideae has been characterized by comparative cytogenetic studies of heterochromatin bands and rDNA sites distribution. In the genus *Crotolaria*, different heterochromatin types were observed, suggesting the occurrence of replacement of repetitive DNA families during the genus diversification (Mondin *et al.*, 2007; Mondin and Aguiar-Perencin, 2011; Morales *et al.*, 2011). In *Astragalus*, it was reported variation in number, intensity, and position of CMA bands along the chromosomes, likewise 5S rDNA sites (Baziz *et al.*, 2014), while cultivated species of *Canavalia* showed variations in the rDNA positions (She *et al.*, 2017). In *Lotus japonicus* (Regel) K. Larsen and *L. filicaulis* Durieu, for which three 35S loci have been identified, initially no polymorphism of this type was observed (Pedrosa *et al.*, 2002). Nevertheless, a comparative cytogenetic map built for *Lotus uliginosus* (L.) Schkuhr (2*n* = 12) revealed intra and interspecific chromosomal rearrangements in *L. japonicus*, *L. filicaulis*, and *L. burttii* Borsos. Changes in the number, size, and position of rDNA sites were observed, as well as an intraspecific heteromorphism of the 5S rDNA site in *L. uliginosus* (Ferreira *et al.*, 2012). Comparative analyses have demonstrated variations in the position of 5S and 45S rDNA sites in *Medicago* L. species, giving evidence of new rearrangements throughout the evolutionary history of the genus (Yu *et al.*, 2017). Meanwhile, the genus *Lens* showed variations mainly in 5S sites (Balyan *et al.*, 2002; Fernandez *et al.*, 2005). Although wide cytogenetic variation related to the repetitive fraction of the genome (heterochromatic bands and rDNA sites) has been reported in different Papilionoid genera, there is little information on the degree of intergeneric variability of whole genomes (Schubert and Giang, 2016).

Variation in heterochromatin composition was also observed in *Stylosanthes*. However, only two species showed different number and positions in CMA^+^ bands. Thus, comparing with *Arachis*, this variability of heterochromatin can still be considered low (Silvestri *et al.*, 2015). Interestingly, DAPI^+^ proximal bands were observed only in *S. ruellioides*. This species belongs to the same clade of *Stylosanthes scabra* complex (*S. viscosa*, *S. hamata*, and *S. scabra*) (Marques *et al.*, 2018), that also showed DAPI^+^ bands in a (peri)centromeric location, suggesting that these species are closely related.

### Evolutionary trends in *Stylosanthes* genome

The knowledge about the karyotype organization in *Stylosanthes* contributes to a better understanding of its evolutionary history, which is so far exemplified by the general stability in genome size and chromosome numbers. Here we evaluated the evolution of karyotypic diversity in *Stylosanthes* by comparing the variation in the number of rDNA sites and DNA content between its sister clade. In general, cytogenetic data give evidence of cytomolecular stability of *Stylosanthes* in relation to *Arachis* (Vieira *et al.*, 1993; Silvestri *et al.*, 2015), suggesting that the age of the group could be an important factor to be considered in karyotype diversification. However, a more representative sampling within *Stylosanthes* and *Arachis* in the dating analysis would be required to confirm the age estimates of major lineages within these genera. Low morphological differentiation, phylogenetic similarity of plastid and nuclear DNA sequences, as well as crosses between species (allopolyploidization) from different clades support the cytomolecular stability in the recently evolved *Stylosanthes* (Vander Stappen *et al.*, 2003). In contrast, *Arachis* presents a high variability of the CMA/DAPI banding and rDNA sites distribution (5S and 18S), being possible to identify six genomes (A, B, D, F, G, and K) (Fernández and Krapovickas, 1994; Peñaloza and Valls, 2005; Silvestri *et al.*, 2015; Ortiz *et al.*, 2017). Smartt *et al.* (1978) initially established the A and B genomes based on the two different chromosome complements in the allotetraploid *A. hypogaea* (AABB). The “A chromosomes” have a differential condensation pattern during prometaphase (Fernández and Krapovickas, 1994) and have a large heterochromatic band DAPI^+^ in the centromeric region (Seijo *et al.*, 2004). The remaining species with symmetric karyotypes, but without “A chromosomes”, have been assigned to one single genome group named B genome or non-A genome (Seijo *et al.*, 2004; Robledo and Seijo, 2010). In addition, *Arachis* presents a greater diversity of species and morphological variability compared to *Stylosanthes*.

Although rDNA occupies a large fraction of the nuclear genome, it is also an unstable genomic region and the reasons for this instability are not fully understood (Kobayashi, 2008; Totta *et al.*, 2017). Recent studies have explored the dynamics of rDNA loci under an explicit phylogenetic framework. Totta *et al.* (2017) inferred that a stasis in 45S rDNA site number occurred during most of the evolutionary history of *Cistus* (Cistaceae) and allied genera. The authors suggested that most of the multiple shifts involving changes in the number of the rDNA loci likely occur since the Middle Pleistocene in this group, and that rDNA stasis in site number may have been underestimated in diploids. The terminal positions of 35 rDNA loci might also facilitate higher frequencies of interlocus homogenization than is found in interstitial or pericentromeric loci (Garcia *et al.*, 2017)

Analysis of the evolutionary rates of DNA sequence data in the rainforest tree genus *Swartzia* (Papilionoideae) indicates that it diversified rapidly after its origin, probably during the Miocene (Torke, 2006). Such information, coupled with cytogenetic features (small chromosomes, two 5S, and 45S rDNA sites in metaphases as well as two positive CMA bands) are preliminary evidences of strongly conserved karyotypes in a recently evolved species-rich lineage (Pinto *et al.*, 2016). The stability observed in some groups of recent divergence was also identified in other papilionoid genera such as *Phaseolus* (Fonsêca and Pedrosa-Harand, 2013, 2017). The lupin genus (*Lupinus*) encompasses about 270 annual and perennial species distributed in both the Old World (generally around the Mediterranean basin) and the New World (primarily North and South America) (Drummond *et al.*, 2012b). The Old World lineage originated between 17–20 Mya while New World species evolved around 2–5 Mya. The Old Word lupins show a high level of genomic diversification characterized by variation in chromosome numbers (2*n* = 32–52), basic chromosome numbers (*x* = 5–7, 9, 13), nuclear genome size, patterns of chromatin modifications at the chromosomal level, and 5S/45S rDNA sites number (Naganowska *et al.*, 2002, 2003; Hajdera *et al.*, 2003; Susek *et al.*, 2016, 2017). On the other hand, New World lupins have more uniform karyotypes in terms of their genome structure, and have chromosome numbers of either 2*n* = 36 or 2*n* = 48 with a fixed basic chromosome number *x* = 6 (Naganowska *et al.*, 2006; Susek *et al.*, 2016). This reinforces the view that within the same group, lineages with older diversification tends to show high karyotype diversity compared to more recent lineages, as was reported here for the sister genera *Arachis* and *Stylosanthes*.

## Supplementary material

The following online material is available for this article:

Table S1 Information about *Stylosanthes* species.

Figure S1 Phylogenetic analysis based on ITS.
