# Supplementary material for: Low cytomolecular diversification in the genus Stylosanthes Sw. (Papilionoideae, Leguminosae)
Source: Genet Mol Biol. 2020 Mar 6;43(1):e20180250. doi: 10.1590/1678-4685-GMB-2018-0250 (PMC7197990; doi:10.1590/1678-4685-GMB-2018-0250)
Supplement: Supplementary file 1 [file 1415-4757-GMB-43-1-e20180250-s001.pdf]

Supplementary Material to "Low cytomolecular diversification in the genus Stylosanthes Sw. (Papilionoideae, Leguminosae)"

Table S1 - Registered number of *Stylosanthes* species.

Species	Number of collection	Number of individuals
S. acuminata M.B.Ferreira & Sousa Costa	CPAC 5270	8
S. gracilis Kunth	CPAC 5615/ BRA- 033502	7
S. grandifolia M.B.Ferreira & Sousa Costa	CPAC 5338/ BRA- 041955	8
S. guianensis (Aubl.) Sw. 1463	GC Grof 1463/ 34879 [a]	7
S. guianensis (Aubl.) Sw. 1480	GC Grof 1480/ 34880[a]	10
S. guianensis (Aubl.) Sw. 4171	CPAC 4171	9
S. guianensis (Aubl.) Sw. LC2538	LC2538	9
S. guianensis (Aubl.) Sw. Mineirão	BRS Mineirão/ 02514[a]	10
S. hippocampoides Mohlenbr	CPAC 5253	8
S. macrocephala M.B.Ferreira & Sousa Costa	CPAC 2235/ BRA- 022516	7
S. ruellioides Mart. ex Benth.	CPAC 2319	7
S. pilosa M.B.Ferreira & Sousa Costa	CPAC 5246/ BRA- 043231	7

[a]Accessions registered in Ministério da Agricultura Pecuária e Abastecimento

CPAC- Centro de Pesquisa Agropecuária dos Cerrados- Embrapa Cerrados (Brazilian Agricultural Research Corporation)
