# Supplementary material for: Low cytomolecular diversification in the genus Stylosanthes Sw. (Papilionoideae, Leguminosae)
Source: Genet Mol Biol. 2020 Mar 6;43(1):e20180250. doi: 10.1590/1678-4685-GMB-2018-0250 (PMC7197990; doi:10.1590/1678-4685-GMB-2018-0250)
Supplement: Supplementary file 2 [file 1415-4757-GMB-43-1-e20180250-s002.pdf]

Supplementary Material to "Low cytomolecular diversification in the genus Stylosanthes Sw. (Papilionoideae, Leguminosae)"

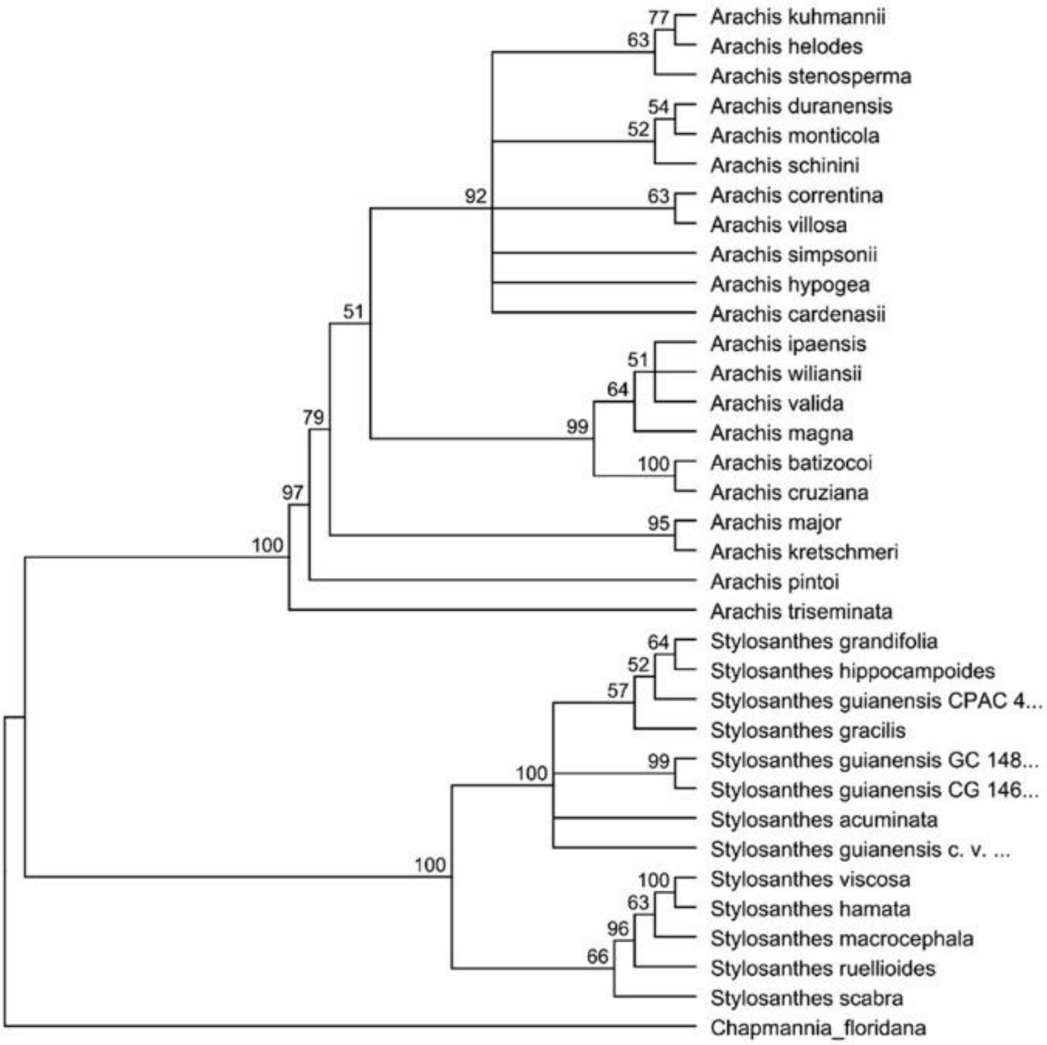

Figure S1- Phylogenetic analysis based on ITS.
